# Detection of *Babesia bovis* using loop-mediated isothermal amplification (LAMP) with improved thermostability, sensitivity and alternative visualization methods

**DOI:** 10.1038/s41598-023-29066-1

**Published:** 2023-02-01

**Authors:** Apinya Arnuphapprasert, Yudhi Ratna Nugraheni, Aung Aung, Masahito Asada, Morakot Kaewthamasorn

**Affiliations:** 1 Veterinary Parasitology Research Unit, Department of Pathology, Faculty of Veterinary Science, Chulalongkorn University, Bangkok, Thailand; 2 Veterinary Pathobiology Graduate Program, Faculty of Veterinary Science, Chulalongkorn University, Bangkok, Thailand; 3 The International Graduate Program of Veterinary Science and Technology (VST), Faculty of Veterinary Science, Chulalongkorn University, Bangkok, Thailand; 4 Department of Parasitology, Faculty of Veterinary Medicine, Universitas Gadjah Mada, Yogyakarta, Indonesia; 5 National Research Center for Protozoan Diseases, Obihiro University of Agriculture and Veterinary Medicine, Obihiro, Japan

**Keywords:** Microbiology, Molecular biology, Zoology, Medical research, Molecular medicine

## Abstract

Bovine babesiosis is one of the most economically important tick-borne diseases in tropical and subtropical countries. A conventional microscopic diagnosis is typically used because it is inexpensive and expeditious. However, it is highly dependent on well-trained microscopists and tends to be incapable of detecting subpatent and chronic infections. Here, we developed a novel nucleic acid-based amplification method using loop-mediated isothermal amplification (LAMP) in conjunction with a colori-fluorometric dual indicator for the rapid and accurate detection of *Babesia bovis* based on the mitochondrial *cytochrome b* gene. We aimed to improve the thermostability, sensitivity, specificity, and alternative visualization of LAMP-based methods. We assessed its diagnostic performance compared to two conventional PCR agarose gel electrophoresis (PCR-AGE) methods. The thermostability of LAMP reaction mixtures and DNA templates in variable conditions was also assessed. In addition, we evaluated alternative visualization methods using different light sources including neon, LED, and UV lights. We found that the LAMP-neon was ten times more sensitive than the PCR-AGE, while the LAMP-LED and LAMP-UV were 1,000 times more sensitive. The current LAMP method showed no cross-amplification with uninfected cattle DNA or other common blood parasites in cattle, including *Babesia bigemina*, *Theileria orientalis*, *Anaplasma marginale*, and *Trypanosoma evansi*. In addition, the developed LAMP method has good thermostability and the potential for on-site utility as *B. bovis* DNA could still be detected up to 72 h after initial preparation. Our findings suggested that the developed LAMP method provides an alternative approach for *B. bovis* detection with sensitivity higher than PCR-AGE diagnostics, high specificity, and the flexibility to use neon, LED, and UV light sources for positive signal observations.

## Introduction

*Babesia bovis* and *Babesia bigemina*, which belong to the Apicomplexa phylum, are two of the most common intraerythrocytic piroplasms that cause bovine babesiosis worldwide^1^. Babesiosis is a tick-borne disease in cattle that infects and destroys healthy erythrocytes, resulting in mortality and morbidity^2^. These parasites cause major economic losses to cattle farm productivity^3,4^. Clinical signs of bovine babesiosis include fever, pale mucous membranes, jaundice, and hematuria. Severe neurological signs and death have been reported, particularly in *B. bovis* infections, which are more virulent than *B. bigemina* infections^3,5^. Notably, the World Organization for Animal Health (WOAH, formerly OIE) included bovine babesiosis on its list of major diseases^6^.

Currently, a vaccine for bovine babesiosis has not been widely accessible to all stakeholders in need, and control of the disease has not been completely successful as a consequence^7^. Rapid diagnosis and prompt treatment are crucial for disease control and the minimization of economic losses^8^. However, a conventional diagnosis is time-consuming and labor-intensive, especially in latent and chronic infections. Identification of the causative agent through microscopic examination, nucleic acid-based diagnostics, and serological tests have been suggested by the WOAH^6^. However, these methods require either expert technicians or expensive equipment. Loop-mediated isothermal amplification (LAMP) is a relatively simple isothermal method that requires a constant temperature and one enzyme to complete the reaction^9^. Several LAMP methods have been developed to detect disease agents in animals, such as bovine babesiosis, as well as many other critical human diseases including leishmaniasis and malaria^10–13^. For instance, Iseki et al.^14^ developed a multiplex LAMP method to detect the rhoptry-associated protein-1 genes of *B. bovis* and *B. bigemina*, while Lie et al.^10^ used LAMP to target the internal transcribed spacer region of *B. bovis* and *B. bigemina*. However, most LAMP assays for the detection of bovine babesiosis use gel electrophoresis and are reliant on cold chain storage, which is inconvenient during field investigation. Thus, a more user-friendly (with less complexity, less reliance on laboratory equipment, and shorter processing time), sensitive, specific, and portable LAMP method is required, and a tool that meets these requirements is believed to be a preferable diagnostic of choice for *B. bovis* detection in field settings. Visualization of the LAMP-based method using portable light-emitting diode (LED) and ultraviolet (UV) light sources in conjunction with colorimetric indicators or fluorescent nucleic acid staining dye makes the test more suitable to use in an on-site setting^15^. Until recently, however, no studies had been conducted on the combination of both indicators, called colori-fluorometric dual indicators (CFI), for the detection of bovine babesiosis.

We developed a novel approach to the LAMP-based method coupled with the colori-fluorometric dual indicator for the rapid and accurate detection of *B. bovis* based on the mitochondrial *cytochrome b* gene. We aimed to improve the thermostability, sensitivity, specificity, and alternative signal visualization of LAMP-based methods. Furthermore, we evaluated the diagnostic performance under various setting conditions, as well as the possibility of cross-amplification with other bovine blood-borne pathogens. Visualizations of LAMP products under LED and UV light sources were proven to be 1,000 times more efficient than PCR-AGE. The current method might be useful for the detection of *B. bovis* and offers alternative approaches for on-site utility (portability).

## Results

### Optimal conditions for LAMP incubation temperatures and times for *Babesia bovis* detection

Incubation temperatures ranging from 59, 61, 63, 65, to 67 °C and incubation times of 45, 60, and 75 min were evaluated. LAMP-positive reactions were observed at 61, 63, and 65 °C after 45, 60, and 75 min of incubation, respectively (Figure S1, left panel). We observed a clear distinction in color and brightness changes between positive and negative results when incubation times were 45 and 60 min. However, no reaction occurred during incubation at 67 °C. Incubation of the reaction for 75 min in the no-template negative control seemed to make the nonspecific amplification visible. The results observed after agarose gel electrophoresis (LAMP-AGE) were consistent with observations by the naked eye using neon, LED and UV light sources (Figure S1, right panel).

### Optimal conditions for the concentrations of dNTPs, primer ratio, MgSO_4_, betaine, and Bst polymerase in LAMP reactions

Different concentrations of dNTPs ranging from 1.2, 1.4, 1.6, to 2.0 mM were tested. Among these, the concentrations of dNTPs at 1.2, 1.4, and 1.6 mM provided a clear distinction between positive and negative results, while there was no sign of reaction at 2.0 mM (Figure S2A). We differentiated the colors of the positive and negative reactions as blue and purple, respectively. The brightness of LED and UV light showed an apparent difference between the positive and negative controls. As observed by the naked eye, MgSO_4_ concentrations at 6 and 8 mM exhibited positive results visualized under LED and UV light (Figure S2B). At 4 and 10 mM of MgSO_4,_ we found that the color changed to blue, which was a false positive. For the effects of using primer ratios at 1:3, 1:4, and 1:5, no ratios showed any difference in brightness (Figure S2C). In this study, we tested betaine concentrations ranging from 0.4, 0.6, and 0.8 to 1.6 M. We observed optimal concentrations at 0.4 and 0.6 M as visualized through neon, LED, and UV light sources (Figure S2D). For the effect of *Bst* polymerase concentrations, we found that the concentration was suitable at 4 units per reaction (Figure S2E). At 8 units per reaction, however, there was a false positive result.

### Optimal formulation for detection of *Babesia bovis*

Formulation 1 showed a slight difference between the positive and negative results after adding CFI and was visualized by the naked eye through neon light (Figure S3A, top left). Formulation 2 demonstrated a distinct difference in color between the positive and negative results and was visualized by the naked eye through neon light (Fig. 3B, top left). However, the brightness of both reactions did not differ when visualized under LED and UV light sources (Figs. 3A,B, middle and bottom left). The results of agarose gel electrophoresis corresponded to those of the reaction tube visualizations (Figs. 3A,B, right). Therefore, we chose Formulation 2 to further evaluate the field samples.

### Sensitivity and specificity of the current LAMP protocol compared to the PCR-AGE assay

**Figure 1:**
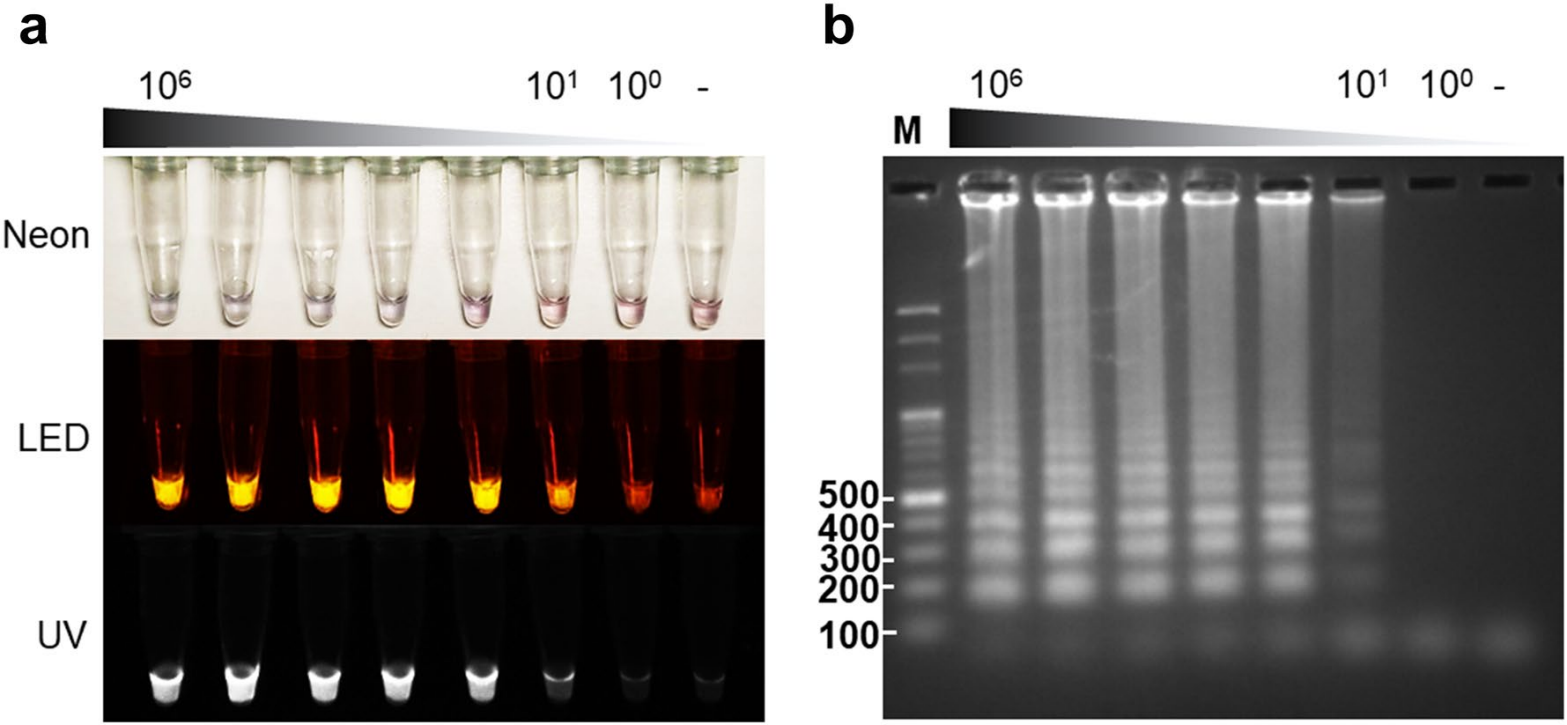
Sensitivity of the LAMP reaction. Serial dilutions of the DNA template were made to determine the detection limit of the LAMP reaction ranging from 10^6^ copies/µL to 10^0^ copies/µL. Visualizations of the LAMP results were made through neon, light-emitting diode, and UV light sources **(A)**. LAMP-agarose gel electrophoresis (LAMP-AGE) photos **(B)** Smear bands indicate positive results. Negative control (−), M = DNA ladder marker. The brightness and contrast of the objects and background were adjusted to facilitate viewing.

**Figure 2:**
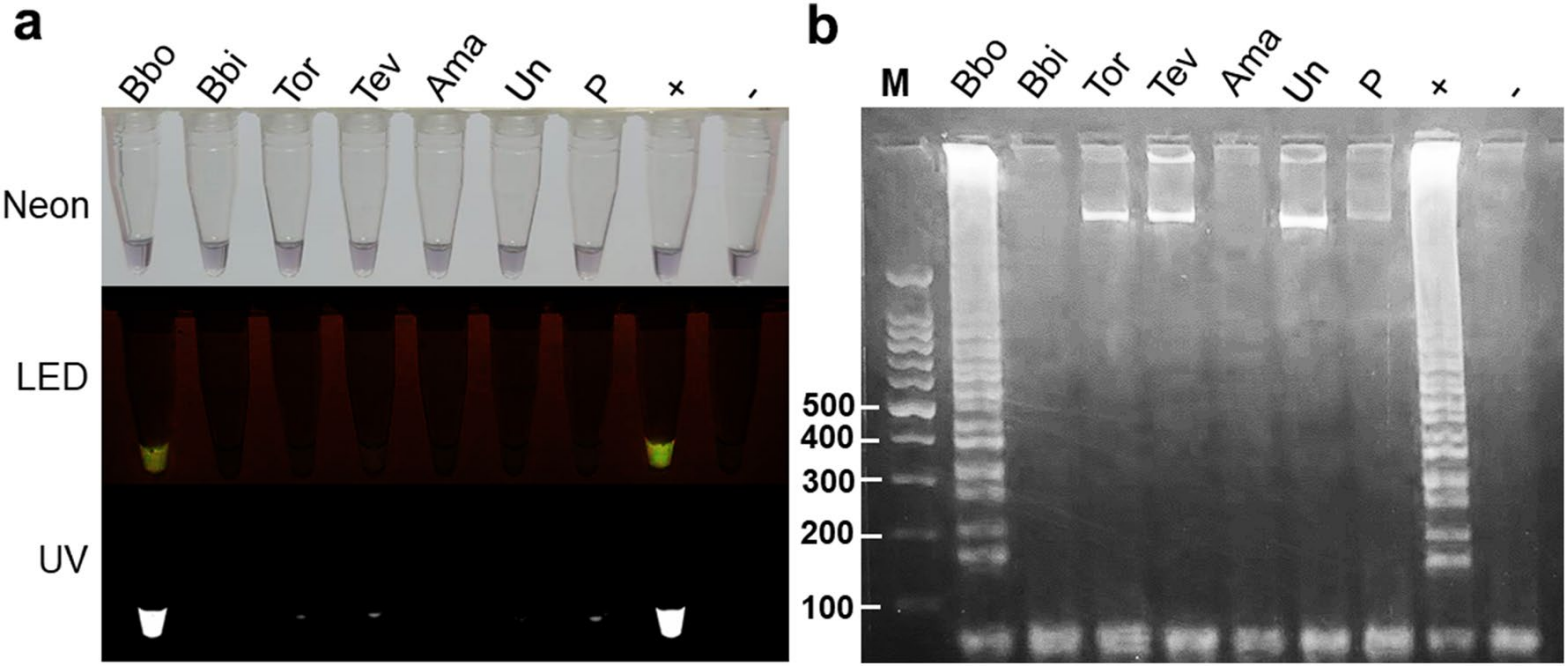
Specificity of LAMP reactions. The potential cross-amplification of the present LAMP protocol was determined using a wide range of DNA templates. Bbo = *B. bovis*, Bbi = *B. bigemina*, Tor = *T. orientalis*, Tev = *T. evansi*, Ama = *A. marginale*, Un = uninfected cattle DNA, P = self-ligated plasmid backbone (pGEM®-T vector), +  = positive control,—= negative control. Visualization of LAMP results from different light sources: Neon, LED, and UV **(A)**. The LAMP-AGE image **(B)**. The brightness and contrast of the objects and background were adjusted to facilitate viewing.

We observed a positive result using neon when the concentration of DNA templates was 10^3^ copies/µL (Fig. 1A). The limit of detection from LAMP-LED and LAMP-UV light revealed 10^1^ copies/µL (Fig. 1A), as well as by electrophoresis (LAMP-AGE) (Fig. 1B). According to these findings, the LAMP-neon was ten times more sensitive than the PCR-AGE, while the LAMP-LED and LAMP-UV were 1,000 times more sensitive (Fig. 1, Figure S4). No cross-reaction of the LAMP assay was observed in the specificity assessment (Fig. 2).

In DNA spiked samples, an internal PCR control using the same LAMP outer primers could detect all true positive and true negative results (Table 1). The McNemar test revealed no statistical significance (*p* = 1), indicating that there were no differences in the results of the PCR-AGE assay and the reference method (external control of PCR). Cohen’s kappa indicated nearly perfect agreement (Cohen’s kappa coefficient = 1). Furthermore, the current PCR-AGE protocol showed 100% sensitivity and specificity in both statistical analyses (Table 1).

**Table 1:** Summary of the performance of LAMP targeting the *B. bovis cytb* gene compared to PCR-AGE methods.

Method	Visualization		External PCR (Romero-Salas et al.^41^)			McNemar’s Test	Cohen’s	Sensitivity	Specificity
			Pos	Neg	Total	*p*—value	kappa	(%)	(%)
Internal PCR	UV light	Pos	33	0	33				
control	(Gel	Neg	0	66	66	1	1	100	100
	electrophoresis)	Total	33	66	99				
	Neon light	Pos	6	0	6				
		Neg	27	66	93	0.00***	0.229	18.2	100
		Total	33	66	99				
	LED light	Pos	28	1	29				
		Neg	3	67	70	0.625	0.904	90.3	98.5
LAMP		Total	31	68	99				
	UV light	Pos	32	0	32				
		Neg	1	66	68	1	0.954	97	100
		Total	33	66	99				
	UV light	Pos	32	0	32				
	(Gel	Neg	1	66	68	1	0.954	97	100
	electrophoresis)	Total	33	66	99				

The bold number with 3 asterisks (***) represents the statistical significance of McNemar’s test as a *p*-value ≤ 0.05. Cohen’s kappa coefficient; < 0 = no agreement, 0–0.20 = slight, 0.21–0.40 = fair, 0.41–0.60 = moderate, 0.61–0.80 = substantial, 0.81–1 = almost perfect agreement.

*Pos.* Positive, *Neg.* Negative, *UV *Ultraviolet, *LED* Light- Emitting Diode.

The McNemar test (*p* < 0.01) suggested statistical significance for visualization with the naked eye using neon light compared to PCR-AGE, while Cohen’s kappa revealed a slightly weak agreement in the results. The sensitivity of the LAMP assay after the addition of CFI was 18.2% and the specificity was 100%. Observing the LAMP results with white LED light revealed no statistical significance (*p* > 0.05) compared to PCR-AGE and were in nearly perfect agreement (K value = 0.90). Furthermore, the sensitivity and specificity were 90.30 and 98.50%, respectively. Under UV light, the LAMP results showed no statistical significance (*p* > 0.05) compared to PCR-AGE and were in nearly perfect agreement (K value = 0.95). The sensitivity and specificity of the current LAMP protocol were 97 and 100%, respectively (Table 1). When observed after agarose gel electrophoresis (LAMP-AGE-UV), all statistical analyses of the LAMP protocol were in agreement with tube-direct visualization under ultraviolet light (LAMP-UV).

### Confirmation of LAMP products

The LAMP products were successfully confirmed by the digestion of restriction enzymes using PCR amplified from the LAMP products as templates, as well as products after being ligated into the plasmid (Figure S5). The LAMP-amplified products had slightly larger fragment sizes than the plasmid reference amplified products, which were used as a positive control in this study. According to the sequencing results, the product was a 100% match to the plasmid template. This evidence indicates that the LAMP method was successful in amplifying the *B. bovis cytb* gene.

### Thermostability of the LAMP reaction for *Babesia bovis* detection

**Figure 3:**
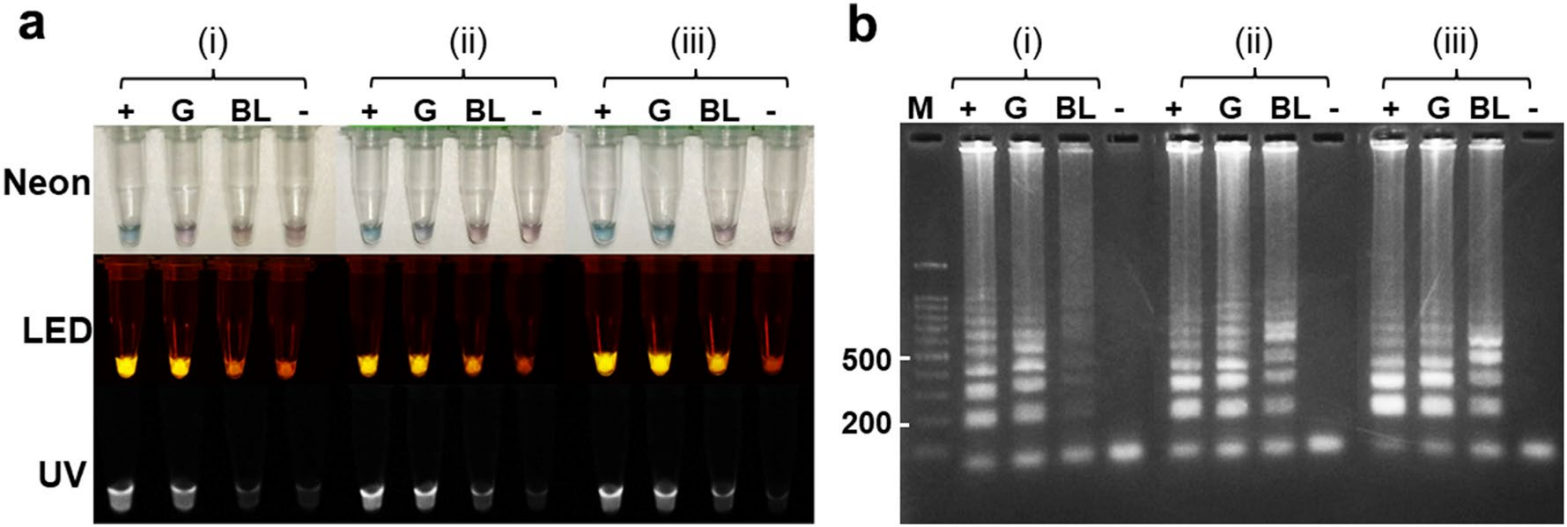
Thermostability of the LAMP reaction after 72 h of storage. Three conditions were evaluated: i) LAMP reaction premixed with *Bst* polymerase and stored at room temperature, ii) LAMP reaction premixed with *Bst* polymerase and stored at 4 °C, and iii) LAMP reaction without *Bst* polymerase, stored at 4 °C, and *Bst* polymerase added just before starting incubation. **(A)** The LAMP reactions were visualized through neon light, LED light, and ultraviolet light. **(B)** agarose gel electrophoresis (LAMP-AGE) photos and smear bands indicate positive results. Abbreviations: ( +) = positive control (pGEMCytbBbo), (G) = gDNA from *B.,* (−) = negative control, (BL) = *B. bovis* infected crude blood*,* and (M) = DNA ladder marker. The brightness and contrast of the objects and background were adjusted to facilitate viewing.

After 12 h of pre-mix preparation, the conditions for three different settings were used to test the reaction performance. A clear signal was observed in the positive control of all the conditions for the environment tested. The color and brightness of the positive and negative results could be differentiated by the naked eye through neon and LED lights after adding CFI, respectively (Figure S6). Nearly identical bands of amplicons were observed after 2% agarose gel electrophoresis. Furthermore, LAMP performance was tested using purified *B. bovis* gDNA and *B. bovis*-infected crude blood samples for reaction mixtures kept for 72 and 168 h after preparation. The LAMP reaction mixture that was kept for 72 h after preparation and stored at room temperature showed a weak signal when visualized through neon light (Fig. 3). After the CFI, the color changed only in the positive control, but a strong difference was observed between the positive and negative controls under LED and UV lights. Furthermore, the results of the LAMP reaction mixture, which was stored at 4 °C with fresh *Bst* polymerase added just before starting incubation, showed a strong positive signal under LED light included with the crude blood sample. Furthermore, the LAMP reaction mixture using freshly added *Bst* polymerase just before starting incubation showed the strongest reaction without false positives (Fig. 3). LAMP reactions were positive under the setting conditions ‘ii and iii’ without false positives in the negative control (Figure S7). Unfortunately, the condition in which the LAMP reaction mixture was stored for 168 h (7 days) after preparation resulted in a false positive.

## Discussion

Several isothermal amplification methods have been developed for identifying infectious agents including rolling circle amplification (RCA), recombinase polymerase amplification (RPA), multiple displacement amplification (MDA), ladder-shape melting temperature isothermal amplification, and helicase-dependent amplification (HDA). These diagnostic methods have been used in the fields of agriculture, medicine, and veterinary medicine (as reviewed by Boonbanjong et al.^16^). Among these, LAMP is one of the most widely used methods. In this study, we successfully developed a LAMP assay targeting the *B. bovis cytb* gene. LAMP products were confirmed by restriction enzyme digestion and sequencing. The pattern generated after the restriction enzyme digestion of the LAMP products revealed similar findings to the study by Kong et al.^17^, which differed slightly from the expected size due to the complexity of the product^9^. The* B. bovis cytb* sequence was confirmed with the LAMP product, suggesting an accurate amplification. The detection limit for the current LAMP assay was 10^1^ copies/µL, which is higher than the conventional PCR-AGE assay (10^4^ copies/µL). According to our findings, the LAMP-neon was ten times more sensitive than the PCR-AGE, while the LAMP-LED and LAMP-UV were 1,000 times more sensitive. Previous studies, such as that of Liu et al.^10^, demonstrated that LAMP targeting the internal transcribed spacer (ITS) of *B. bovis* had a detection limit of 0.1 pg with no cross-amplification to other pathogens. According to the *B. bovis* genome size of 8.2 Mbp^18^, 0.1 pg of DNA is estimated to be equivalent to 12 parasites^19^. Furthermore, Yang et al.^11^ demonstrated that LAMP was 100 times more sensitive than PCR in detecting *B. bovis* (*cytb* gene) when combined with the lateral flow dipstick (LFD). Although the number of *B. bovis cytb* genes per parasite is unknown, PCRs targeting the *cytb* gene have been shown to be more sensitive than those targeting the 18S rRNA gene^20^. The most well-studied apicomplexan parasite, *Plasmodium* spp., has 4–8 copies of the 18S rRNA gene and 20–150 copies of mitochondrial genes per parasite^21,22^. Based on the assumption that *B. bovis* and *Plasmodium* species share a similar number of copies of the *cytb* gene, the current LAMP protocol has the ability to detect *B. bovis* at 0.001 to 0.01% parasitemia, which could determine *B. bovis* infection^23,24^. Extending the incubation time could improve the sensitivity of LAMP^25^. However, it may risk generating false positive signals, as reported in a study by Hardinge and Murray^26^, as well as in this study. The specificity test of the current study reveals no cross-reaction with other vector-borne pathogens that are usually found in cattle, including self-ligation of the plasmid backbone, similar to the results from previous studies^10,11^.

Colorimetric detection in this study showed low sensitivity (18.2%). The Mg^2+^ ion is crucial in changing the color of hydroxy naphthol blue (HNB) from violet to blue sky in positive samples^27^. Furthermore, the low DNA template revealed no distinction for HNB^28^. Several other colorimetric indicators, such as phenol red, malachite green, neutral red, and others, have also been used for LAMP detection^28–30^. In the present study, statistical analysis revealed nearly perfect agreement between visualization through LED and UV lights. In this study, visualization with a fluorescent nucleic acid staining dye such as gel green outperformed the colorimetric indicator. Our findings suggested that dual indicators may be more reliable than colorimetric indicators alone to support the interpretation of the LAMP assay. The current LAMP preparation remained working for a few days, indicating that the method has good thermostability and the potential for on-site utility. Low primer purity is most likely responsible for self-binding and false positives in LAMP assays^26^. Furthermore, nonspecific DNA amplification is partly influenced by temperature^31^. Previously, the method for trypanosome detection by Hayashida et al.^32^ was performed using a dry-LAMP reaction; it was able to maintain the reaction performance for 7 months. The findings of Hayashida et al.^32^ suggest that LAMP in dry form and long-term storage is feasible. The amplification reaction seems to be largely influenced by thermostability and subsequently affects polymerase performance ^33^. Therefore, further optimization of the ready-made LAMP premix for *B. bovis* detection could be beneficial for on-site utility and could reduce the risk of contamination during field preparation. Although this study demonstrated the success of LAMP amplification using purified DNA and 0.1% (v/v) triton X-100 treated crude samples of infected red blood cells, additional studies concerning the field samples are required.

In conclusion, we developed a novel LAMP-based method that uses a dual indicator for visualization to detect *B. bovis*. The protocol was validated, and its performance in detecting *B. bovis* was evaluated. Compared to PCR-AGE methods, the current LAMP assay appears to be more sensitive and highly specific for detecting *B. bovis* infection. We successfully demonstrated alternative visualization methods and found that LAMP-UV appeared to be the most sensitive approach. The current LAMP method could be useful and applicable for detecting clinical and subclinical bovine babesiosis.

## Methods

### DNA samples used in this study

The genomic DNA of the *B. bovis* Texas strain T2Bo was used as a reference. *Babesia bigemina* was kindly provided by Dr. Montakan Jiratanh from the Thailand National Institute of Animal Health (NIAH)^34,35^. *Theileria orientalis, Trypanosoma evansi,* and *Anaplasma marginale* were obtained from our previous studies^36–38^. Negative control was also used, which originated from non-infected cattle that tested negative for *Babesia* spp. A NucleoSpin® Blood Kit (Macherey–Nagel, Germany) was used to extract DNA samples, as previously described by Nguyen et al.^36^. DNA extraction quality was confirmed by PCR targeting endogenous mammal DNA following the protocol described by Birkenheuer et al.^39^.

### Preparation of Plasmid DNA

The mitochondrial *cytb* gene of *B. bovis* was amplified using BboCytBFW and BboCytBRV primers (Table S1). The PCR mixture contained 2.5 µL of 10X Takara buffer, 0.2 mM dNTP, 0.2 mM forward and reverse primers, and 0.625 U Takara Ex Taq DNA polymerase (Takara, Japan). The PCR cycling conditions included the following: Initial denaturation at 94 °C for 2 min, followed by 40 cycles at 94 °C for 20 s, 52 °C for 20 s, and 72 °C for 30 s, followed by 72 °C for 15 min to complete and held at 12^°^ C. The PCR product was loaded onto a 1.5% agarose gel for electrophoresis for 40 min at 100 V and 400 mA. The A-overhang PCR products (1,297 bp) were cut and purified according to the manufacturer’s instructions for the gel according to the Nucleospin® Gel and PCR clean-up kit (Macherey–Nagel, Germany). The purified PCR product was cloned into the pGEM® T vector (Promega, USA). The plasmids were extracted using NucleoSpin® Plasmid EasyPure (Macherey–Nagel, Germany) and sequenced to confirm the nucleotide sequence before being used to evaluate the LAMP primers.

### LAMP’s primer design

**Figure 4:**
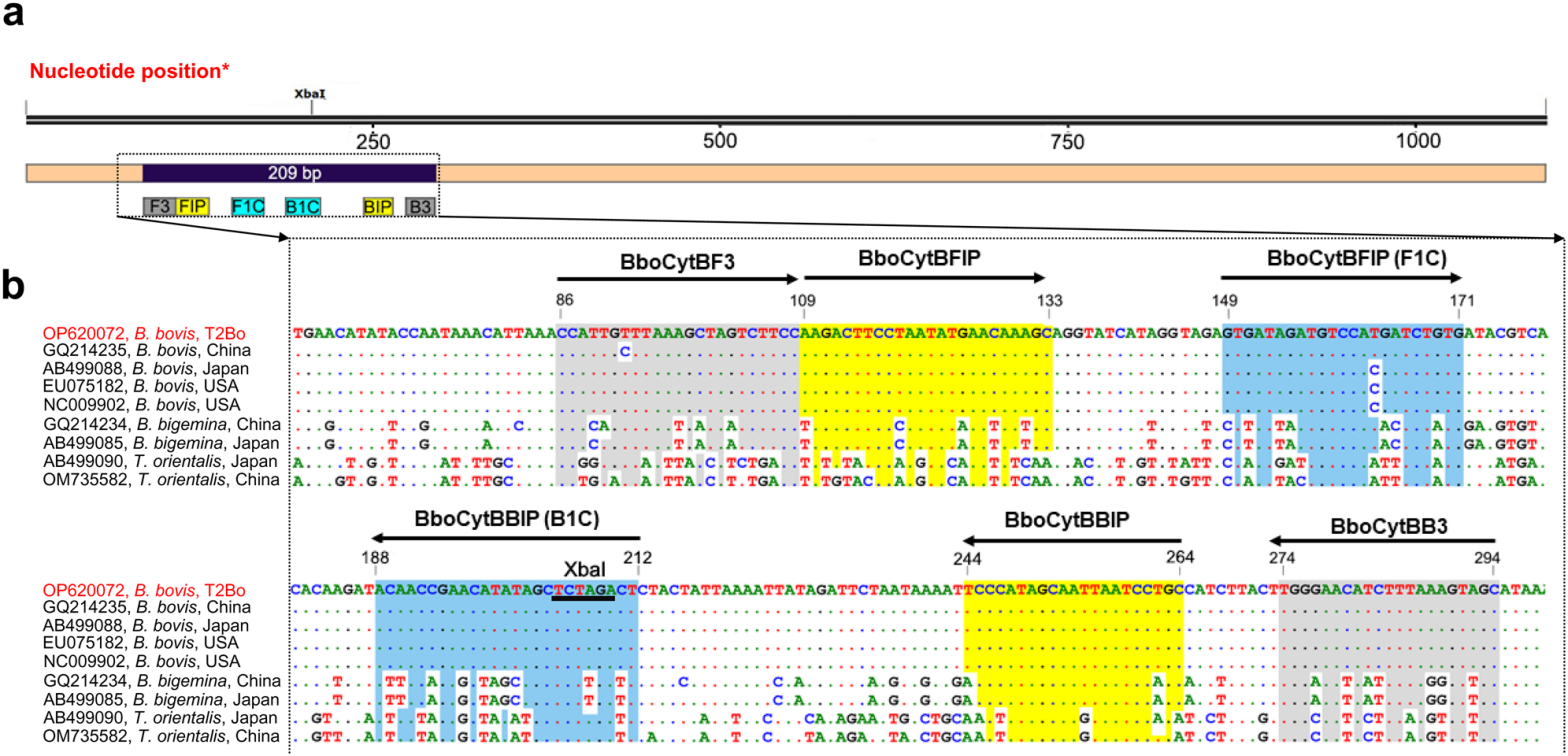
LAMP primer design based on the *cytb* gene of the *B. bovis* Texas T2Bo strain*.* **(A)** Schematic representation of the *B. bovis cytb* sequence depicting the hybridization regions of the primers. The restriction enzyme recognition site is indicated for XbaI. **(B)** ClustalW multiple sequence alignment of *B. bovis* and other parasites in the Piroplasmida order. Nucleotide positions are referred to as the *cytb* gene of the *B. bovis* Texas T2Bo strain (accession no. OP620072) in reverse complement. The outer primers (BboCytBF3 and BboCytBB3) target nucleotide sequence in gray shades, and the inner primers (BboCytBFIP and BbocytBBIP) are yellow and blue shades, respectively. In each primer, the conserved nucleotides and polymorphic sites are presented as dots and characters in colors, respectively. The restriction sequence (XbaI) is underlined.

To identify candidate regions specific for *B. bovis,* nucleotide sequences of Piroplasmida parasites commonly found in cattle, including *B. bovis*, *B. bigemina,* and *Theileria orientalis*, were aligned using BioEdit v7.0.5.3 software v7.0.5.3^40^ (Fig. 4). The LAMP primers were designed to target the *B. bovis cytb* gene using Primer Explorer V5 software (http://primerexplorer.jp/lampv5e/index.html). The candidate primer sets were chosen following the instructions of the primer explorer (Table S1).

### LAMP reaction optimization

LAMP reactions were carried out under different conditions including variable incubation temperature, time, and concentrations of dNTPs, MgSO_4_, primer ratio, betaine, and *Bst* polymerase. The effects of incubation temperatures were evaluated at 59, 61, 63, 65, and 67 °C. Incubation times were 45, 60, and 75 min. The dNTP concentrations were 1.2, 1.4, 1.6, and 2 mM. The MgSO_4_ concentrations were 4, 6, 8, and 10 mM. The outer-to-inner primer ratios were 1:3, 1:4, and 1:5. The betaine concentrations (Sigma-Aldrich, Germany) were 0.4, 0.6, and 0.8 M. *Bst* polymerase (NEB, USA) was adjusted to 4 and 8 U. All reactions were reproduced at least 3 times.

### Comparison of two LAMP formulations

Two optimal conditions were chosen from the above-mentioned assessments to further evaluate the suitable formulation. The first formulation (referred to as formulation 1) contained 1X buffer, 1.6 mM dNTPs, 8 mM MgSO_4_, a primer ratio of 1:4, 0.6 M betaine, and 4 units of *Bst* polymerase. The second formulation (referred to as formulation 2) contained 1X buffer, 1.4 mM dNTPs, 6 mM MgSO_4_, a primer ratio of 1:4, 0.4 M betaine, and 4 units of *Bst* polymerase. Reactions were incubated for 60 min at 65 °C. Both formulations were evaluated at least 3 times.

### Sensitivity and specificity assessments

The detection limits of LAMP reactions were determined by serial dilutions of the *B. bovis* plasmid template. The concentration was determined using a NanoDrop Lite spectrophotometer (Thermo Fisher Scientific, USA). The DNA copy number was calculated as follows: (DNA amount (ng) × [6.022 × 10^23^])/(length × [1 × 10^9^] × 660). Plasmid DNA templates containing *B. bovis cytb* at concentrations ranging from 10^8^ to 10^0^ copies per µL were evaluated. In this study, DNA samples from various pathogens including *B. bigemina*, *T. orientalis*, *T. evansi*, *A. marginale,* and non-infected cattle were tested for LAMP specificity. Nucleic acid-free sterile distilled water was used as a negative control. The sensitivity and specificity assays were conducted three times.

### Confirmation of LAMP products

The LAMP product was amplified by outer primers (CytbF3 and CytbB3) and then confirmed by restriction enzyme digestion and sequencing, as in previous studies^10,14^. The LAMP product was diluted five times with sterile distilled water and PCR amplified following the PCR protocol described above. The digestion of restriction enzymes was carried out using XbaI (the recognition site is indicated in Fig. 4). Briefly, 10 µL of PCR product, 2 µL of CutSmart buffer, and 0.5 µL of XbaI (NEB, USA) were mixed, and the reaction volume increased to 20 µL with PCR grade water. The reaction mixture was incubated at 37 °C for 60 min before the enzyme was inactivated at 70 °C for 20 min. The digested product was loaded onto a 2% agarose gel and electrophoresed at 100 V for 30 min. The agarose gel was stained with ethidium bromide, and the cutting fragments were visualized under UV transillumination. The 200-bp band was excised from agarose gel, purified, and cloned into the pCR2.1 TOPO vector before sequencing. Sequencing was carried out using the Applied Biosystem 3130xl Genetic Analyzer (Thermo Fisher Scientific, USA). To confirm the nucleotide sequence of the LAMP product, the obtained sequencing results were subjected to a BLASTn similarity search in the GenBank database.

### Thermostability of LAMP reaction mixtures

The thermostability of the LAMP reaction mixture was evaluated under three conditions: i) reaction premixed with *Bst* polymerase and stored at room temperature (20—25 °C) before testing, ii) reaction premixed with *Bst* polymerase and stored at 4 °C, and iii) reaction mixture without *Bst* polymerase initially (polymerase was added just before assessment) and stored at 4 °C. The LAMP mixtures were tested after 12, 76, and 168 h of storage. To ensure reproducibility, we repeated the thermostability tests three times.

## *Babesia bovis* DNA spike and DNA templates in variable conditions

We used a double-blinded method, and all technicians who prepared the DNA template and carried out the LAMP experiment were independent. Thirty-three non-infected cattle DNA samples out of 99 were randomly spiked with 10^7^ copies/µL final concentration of *B. bovis* plasmid. Furthermore, different conditions of DNA templates were tested in this experiment, including purified *B. bovis* DNA and 0.1% (v/v) triton X-100 treated *B. bovis*-infected crude blood.

### Visualization of LAMP reactions

A total of 3 µL of Colori-Fluorometric Dual Indicator (CFI) containing 3 mM of hydroxy naphthol blue, (HiMedia Laboratories, India) and 0.35% v/v of GelGreen (Biotium, USA) was added to the LAMP product tube according to Hayashida et al.^32^. To investigate an alternative visualization method for the LAMP reaction, inspection tools including i) Neon light, ii) LED white light/blue light with an orange ultraviolet filter, iii) UV light with an orange ultraviolet filter, and iv) UV light with an orange UV filter observed after gel electrophoresis were assessed. LAMP reaction tubes in room light (neon light) that remain purple when observed by the naked eye (i) are interpreted as negative. LAMP products that turn from purple to blue are indicative of positive. With white light/blue light and observation through an orange UV filter (ii), the LAMP positive reaction tubes turn into green-fluorescent bright light. Due to equipment availability during the experiment, the validation and specificity test results were visualized using a BluePAD Dual LED Blue/White Light Transilluminator (BIO-HELIX—BP001CU, Taiwan), while the sensitivity and thermostability test results were visualized using a MastroGen UltraSlim LED Transilluminator SLB-01W (MastroGen, Taiwan) with blue light (see equipment and brightness set-up in Figure S8). UV-illuminated LAMP reaction tubes (iii) are interpreted as positive. Finally, smear bands under ultraviolet (UV) light (iv) are interpreted as positive in LAMP products that have undergone agarose gel electrophoresis. Images were taken using the Vü-F fluorescence imaging system (Pop-Bio, UK) for UV visualization. Adobe Photoshop (version 24.0) was used to make brightness and contrast adjustments to the images.

### Independent PCR-AGE methods for *Babesia bovis* detection compared to the current LAMP method

The diagnostic performance of a newly-established LAMP detection method for *B. bovis* was compared with two independent PCR-AGE assays. The first PCR-AGE assay served as an internal control using the same outer primers as the current LAMP assay (BboCytBF3 and BboCytBB3). The second PCR-AGE protocol served as an external control and was carried out according to Romero-Salas et al.^41^. Briefly, the total volume of the PCR mixture was 12.5 µL including 1X Takara buffer solution, 0.2 mM of dNTPs, 0.2 µM/µL of each forward and reverse primers, 1 µL of DNA template, and 8.625 µL of sterile distilled water. The PCR setting consisted of one cycle of initial denaturation at 98 °C for 2 min, followed by 98 °C for 10 s, 52 °C for 15 s, 72 °C for 20 s for 30 cycles and one cycle of final extension at 72 °C for 5 min.

### Statistical Analyses

The diagnostic performance of PCR-AGE obtained from the method of Romero-Salas et al.^41^ was used as a reference (external control). The diagnostic performance of the PCR-AGE and LAMP methods was evaluated for association and agreement using the McNemar test and Cohen’s kappa, respectively. McNemar’s test with a *p*-value < 0.05 was considered to be statistically significant. Cohen’s kappa coefficient < 0 was considered to be no agreement, followed by 0–0.20 slight, 0.21–0.40 fair, 0.41–0.60 moderate, 0.61–0.80 substantial, and 0.81–1 nearly perfect agreement, respectively. Furthermore, the sensitivity (true positive/sum of true positive and false negative) and specificity (true negative/sum of false positive and true negative) were evaluated. All statistical analyses in this study were performed using IBM SPSS Statistics 22; statistical significance was considered when the *p*-value < 0.05.

### Ethical statement and biosafety

This study has been approved by the Institutional Animal Care and Use Committee of the Faculty of Veterinary Science, Chulalongkorn University (IACUC No. 2031098). All experiments were carried out according to the Institutional Biosafety Committee (IBC No. 2031044) and university policies and regulations. This study was reported in accordance with ARRIVE guidelines (https://arriveguidelines.org).

## Supplementary Information

Supplementary Figure 1.

Supplementary Figure 2.

Supplementary Figure 3.

Supplementary Figure 4.

Supplementary Figure 5.

Supplementary Figure 6.

Supplementary Figure 7.

Supplementary Figure 8.

Supplementary Table 9.

## Data Availability

The nucleotide sequences obtained in this study were deposited in the GenBank™ database (https://www.ncbi.nlm.nih.gov/nuccore) under the following accession numbers: OP620072–74 and OP641829–30.

## Abbreviations

LAMP: Loop-mediated isothermal amplification
AGE: Agarose gel electrophoresis
PCR: Polymerase chain reaction
LED: Light-emitting diode
UV: Ultraviolet
CFI: Colori-fluorometric dual indicators
WOAH: World Organization for Animal Health
