# Supplementary figures and images for: Detection of Babesia bovis using loop-mediated isothermal amplification (LAMP) with improved thermostability, sensitivity and alternative visualization methods
Source: Sci Rep. 2023 Feb 1;13:1838. doi: 10.1038/s41598-023-29066-1 (PMC9892585; doi:10.1038/s41598-023-29066-1)

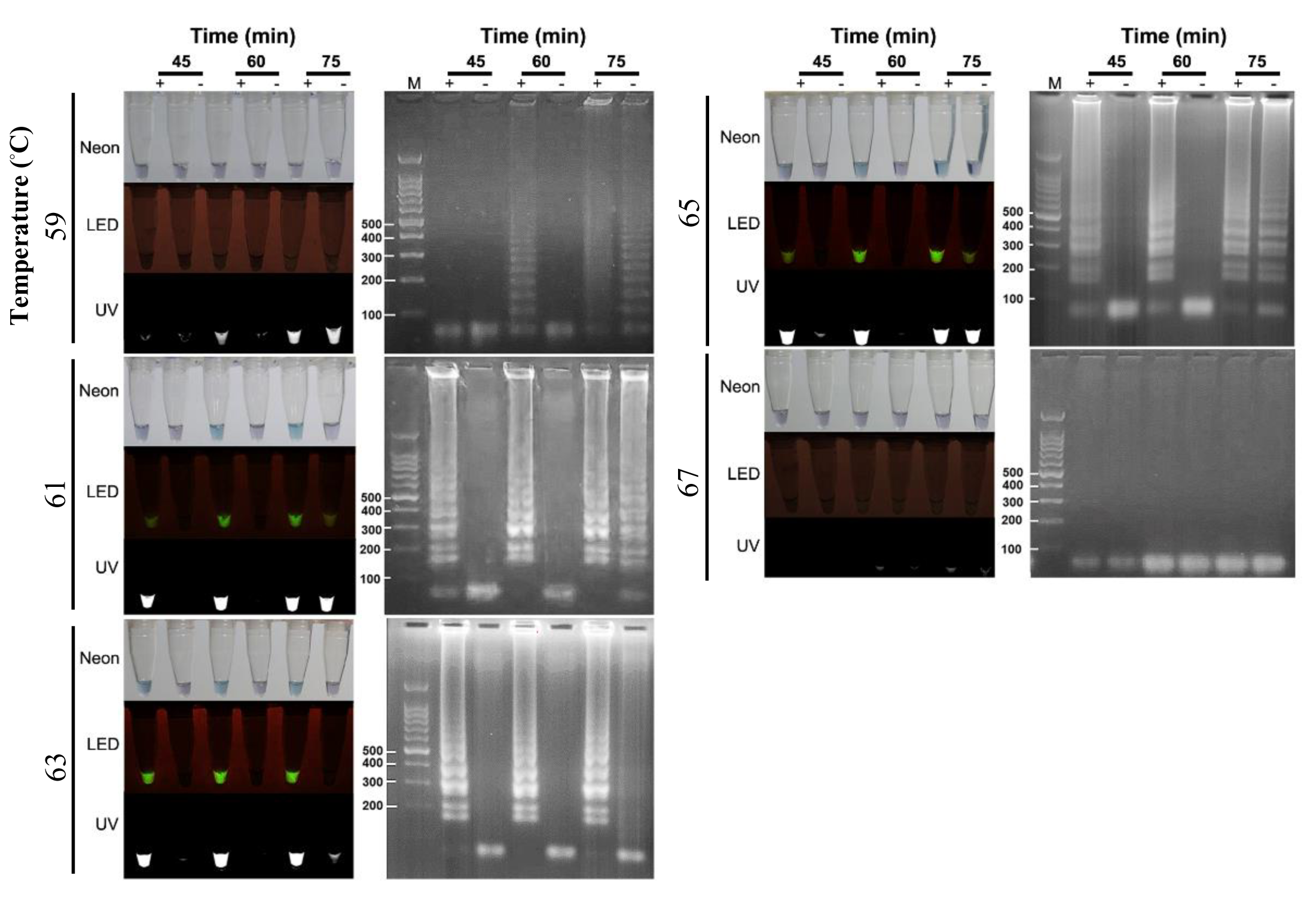

Supplement: Supplementary file 1 — Supplementary Figure 1. [file 41598_2023_29066_MOESM1_ESM.tif]

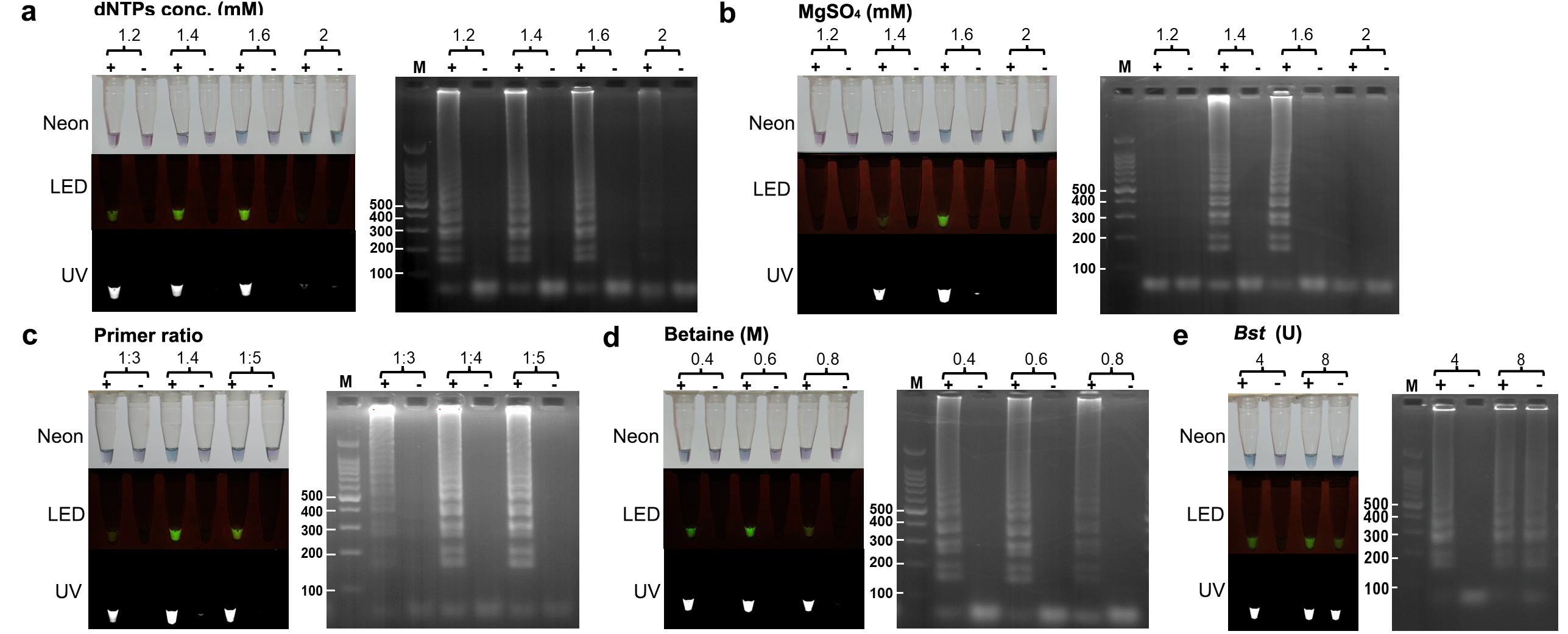

Supplement: Supplementary file 2 — Supplementary Figure 2. [file 41598_2023_29066_MOESM2_ESM.tif]

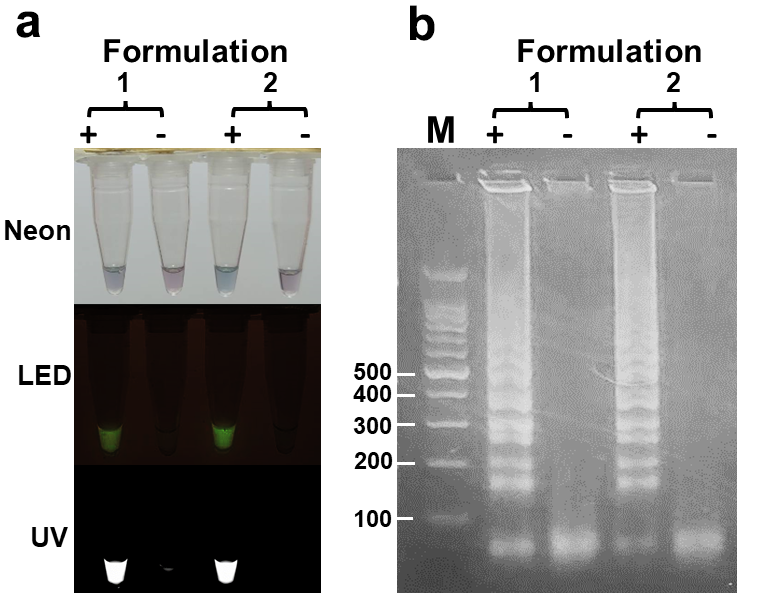

Supplement: Supplementary file 3 — Supplementary Figure 3. [file 41598_2023_29066_MOESM3_ESM.tif]

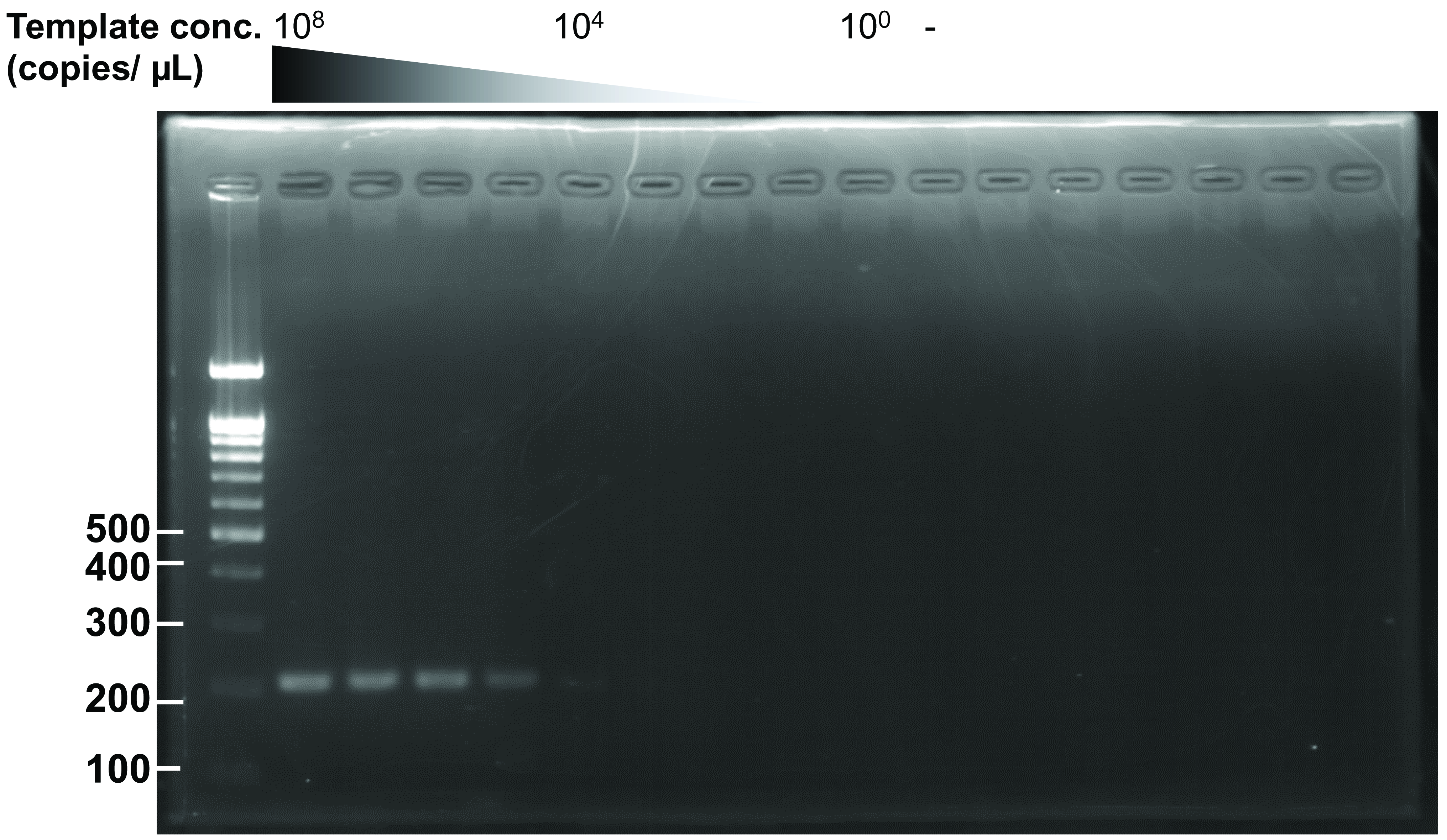

Supplement: Supplementary file 4 — Supplementary Figure 4. [file 41598_2023_29066_MOESM4_ESM.tif]

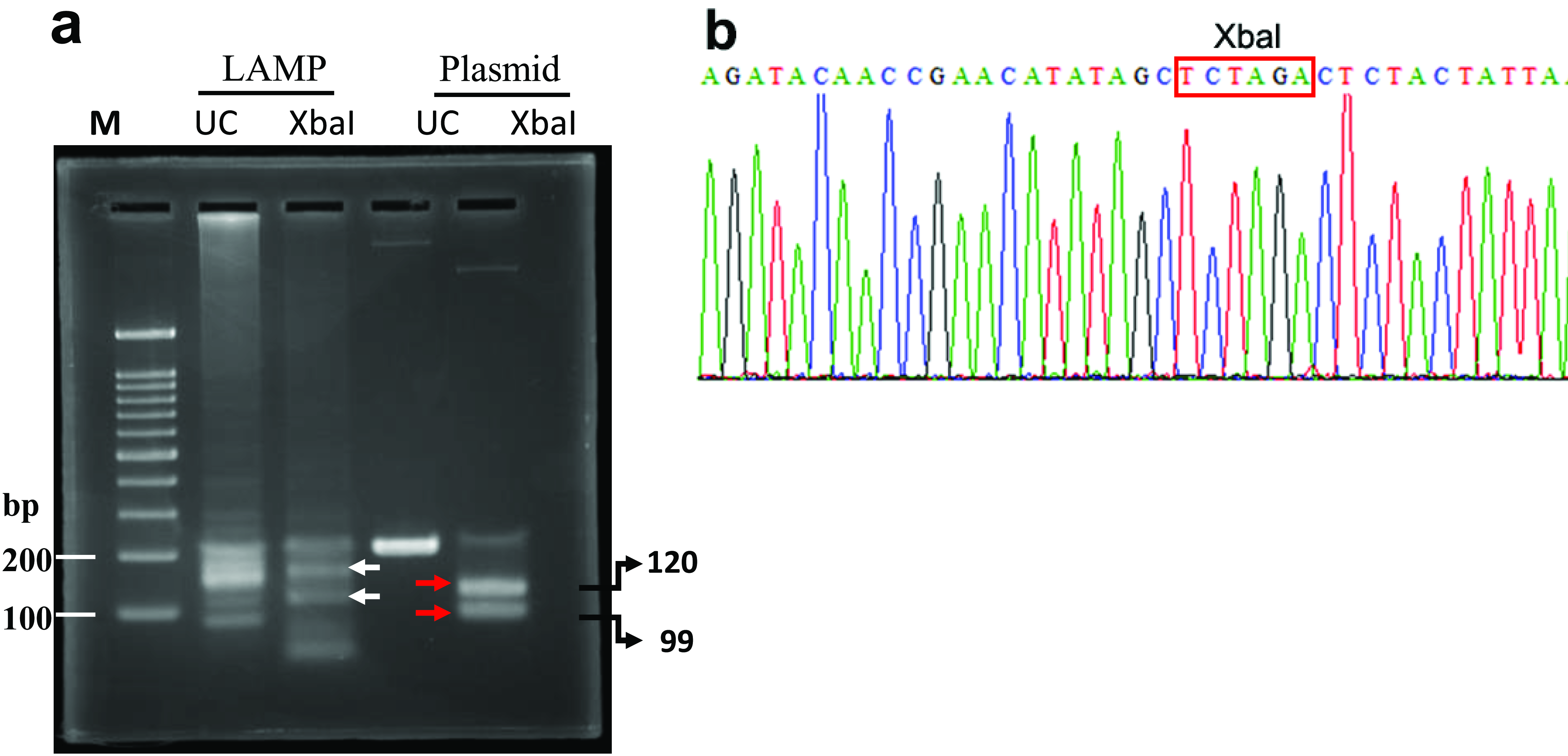

Supplement: Supplementary file 5 — Supplementary Figure 5. [file 41598_2023_29066_MOESM5_ESM.tif]

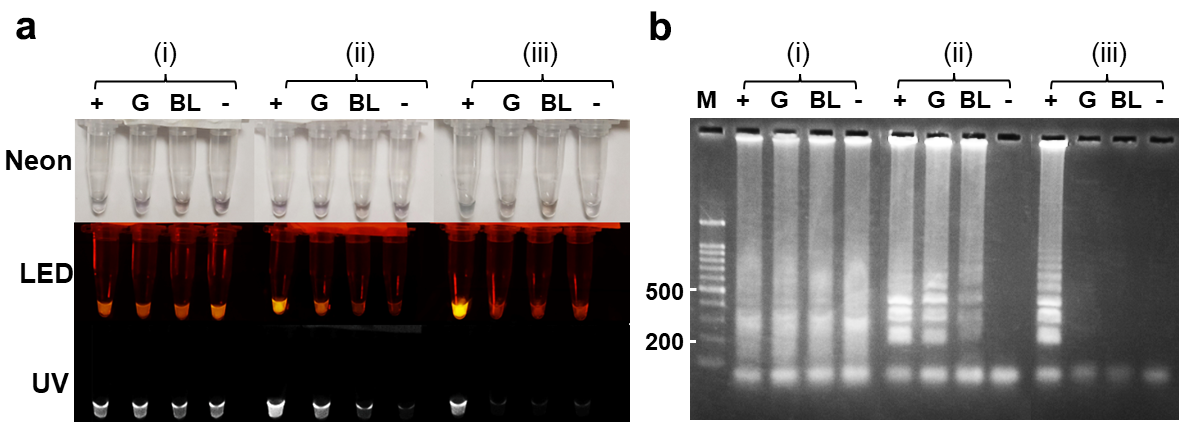

Supplement: Supplementary file 6 — Supplementary Figure 6. [file 41598_2023_29066_MOESM6_ESM.tif]

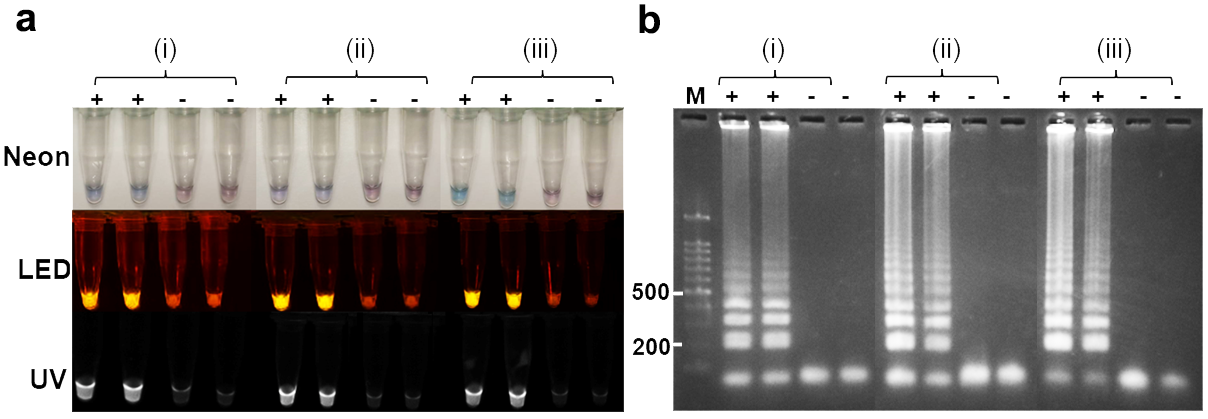

Supplement: Supplementary file 7 — Supplementary Figure 7. [file 41598_2023_29066_MOESM7_ESM.tif]

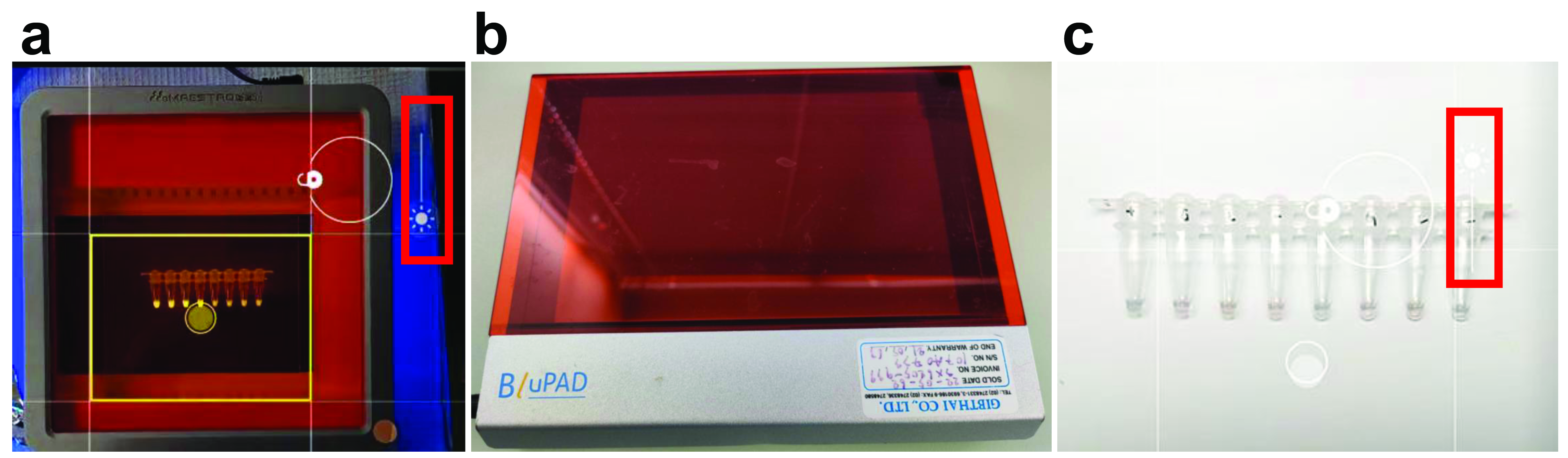

Supplement: Supplementary file 8 — Supplementary Figure 8. [file 41598_2023_29066_MOESM8_ESM.tif]
