# Supplementary material for: Detection of Babesia bovis using loop-mediated isothermal amplification (LAMP) with improved thermostability, sensitivity and alternative visualization methods
Source: Sci Rep. 2023 Feb 1;13:1838. doi: 10.1038/s41598-023-29066-1 (PMC9892585; doi:10.1038/s41598-023-29066-1)
Supplement: Supplementary file 9 — Supplementary Table 9. [file 41598_2023_29066_MOESM9_ESM.pdf]

Table S1 Primers for LAMP and PCR targeting the *B. bovis cytb* gene

**Method**	**Primer name**	**Sequence (5’-3’)**	**Length (bp)**	**Reference**
LAMP	BboCytBF3	CCATTGTTTAAAGCTAGTCTTCC	23	This study
	BboCytBB3	GCTACTTTAAAGATGTTCCCA	21	This study
	BboCytBFIP	CACAGATCGTGGACATCTATCACAAGACTTCCTAATATGAACAAAGC	48	This study
	BboCytBBIP	ACAACCGAACATATAGCTCTAGACTGCAGGATTAATTGCTATGGGA	47	This study
PCR-AGE	BboCytBFWD	ATGATAGCGGTTAATCTTTCCTATTC	26	This study
	BboCytBREV	GAAATTTAGTGAAGGAACTTGACAGG	26	This study
	obo_mit_F	TGAACAAAGCAGGTATCATAGG	22	Romero-Salas et al.[41]
	obo_mit_R	TGAACAAAGCAGGTATCATAGG	22	Romero-Salas et al.[41]
